# Cross-sectional study on smoking types and stroke risk: development of a predictive model for identifying stroke risk

**DOI:** 10.3389/fphys.2025.1528910

**Published:** 2025-03-24

**Authors:** Chao Ding, Minjia Yuan, Jiwei Cheng, Junkai Wen

**Affiliations:** ^1^ Putuo Hospital, Shanghai University of Traditional Chinese Medicine, Shanghai, China; ^2^ Aviation Health Department, Spring Airlines Co.,Ltd, Shanghai, China

**Keywords:** stroke, machine learning, prediction model, Shap, XGBoost

## Abstract

**Background:**

Stroke, a major global health concern, is responsible for high mortality and long-term disabilities. With the aging population and increasing prevalence of risk factors, its incidence is on the rise. Existing risk assessment tools have limitations, and there is a pressing need for more accurate and personalized stroke risk prediction models. Smoking, a significant modifiable risk factor, has not been comprehensively examined in current models regarding different smoking types.

**Methods:**

Data were sourced from the 2015–2018 National Health and Nutrition Examination Survey (NHANES) and the 2020–2021 Behavioral Risk Factor Surveillance System (BRFSS). Tobacco use (including combustible cigarettes and e-cigarettes) and stroke history were obtained through questionnaires. Participants were divided into four subgroups: non-smokers, exclusive combustible cigarette users, exclusive e-cigarette users, and dual users. Covariates such as age, sex, race, education, and health conditions were also collected. Multivariate logistic regression was used to analyze the relationship between smoking and stroke. Four machine-learning models (XGBoost, logistic regression, Random Forest, and Gaussian Naive Bayes) were evaluated using the area under the receiver-operating characteristic curve (AUC), and Shapley’s additive interpretation method was applied for feature importance ranking and model interpretation.

**Results:**

A total of 273,028 individuals were included in the study. Exclusive combustible cigarette users had an elevated stroke risk (β: 1.36, 95% CI: 1.26–1.47, *P* < 0.0001). Among the four machine-learning models, the XGBoost model showed the best discriminative ability with an AUC of 0.794 (95% CI = 0.787–0.802).

**Conclusion:**

This study reveals a significant association between smoking types and stroke risk. An XGBoost-based stroke prediction model was established, which has the potential to improve the accuracy of stroke risk assessment and contribute to personalized interventions for stroke prevention, thus alleviating the healthcare burden related to stroke.

## Graphical Abstract

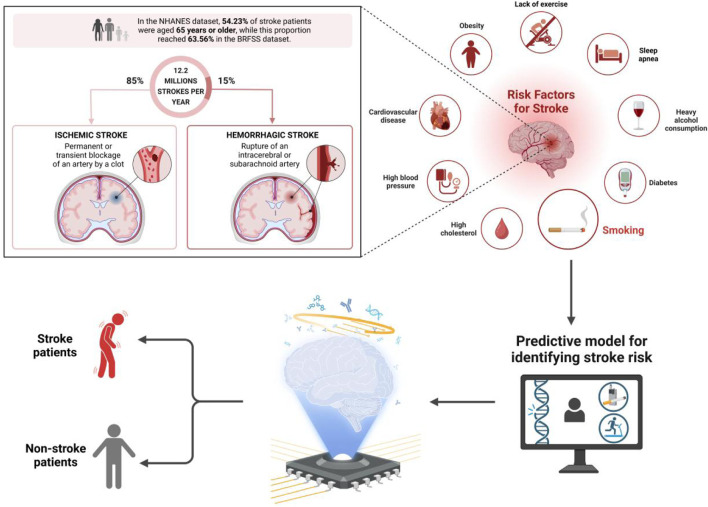

## 1 Introduction

Stroke represents a critical global health challenge, defined by the World Health Organization as the sudden onset of clinical symptoms indicative of focal or global cerebral dysfunction, typically lasting more than 24 h or leading to death, with no apparent cause other than vascular origin (Hilkens et al., 2024). This condition manifests primarily in two forms: ischemic stroke, accounting for approximately 85% of cases, and hemorrhagic stroke, comprising the remaining 15% (Maida et al., 2024; Tu et al., 2023a).

The pathogenesis of ischemic stroke involves the obstruction of cerebral blood flow, predominantly due to atherosclerosis or embolism. In contrast, hemorrhagic stroke results from the rupture of intracranial or peri-cerebral blood vessels, causing hemorrhage and subsequent compression of brain tissue. As a leading cause of mortality and long-term disability worldwide, stroke accounted for over 12.2 million incident cases and 6.55 million deaths in 2020 (Tu et al., 2023a; Ananth et al., 2023; Tu et al., 2023b). The global burden of stroke is projected to escalate further, driven by demographic aging and the increasing prevalence of modifiable risk factors such as hypertension and obesity (Tsao et al., 2023; Martin et al., 2024). This epidemiological trend highlights the critical need for enhanced predictive capabilities to identify high-risk populations and implement preventive measures effectively.

The consequences of stroke extend beyond acute mortality, often resulting in persistent neurological deficits that impose substantial personal and societal burdens, including extensive requirements for long-term care and rehabilitation (Feigin et al., 2023). While multiple risk factors contribute to stroke susceptibility, they can be broadly categorized into modifiable and non-modifiable determinants. Modifiable factors include metabolic disorders (diabetes, dyslipidemia), lifestyle behaviors (smoking, physical inactivity), and dietary patterns, whereas non-modifiable factors encompass genetic predisposition, age, and sex (Tu et al., 2023a; Ekker et al., 2023). Among these, smoking emerges as a particularly potent risk factor, with its impact on stroke risk varying according to smoking type, intensity, and duration (Martin et al., 2024; Wang Y. et al., 2024). Despite the identification of these risk factors, conventional risk assessment tools demonstrate limited predictive accuracy and generalizability across diverse populations.

The advent of artificial intelligence (AI), particularly machine learning (ML) and deep learning (DL) technologies, has revolutionized stroke risk prediction through their capacity to discern complex patterns within extensive datasets (Singh et al., 2024). These advanced computational approaches have demonstrated superior performance in risk stratification compared to traditional methods, enabling more precise and individualized risk assessments. The integration of AI with emerging data sources, including electronic health records and wearable devices, facilitates continuous monitoring and early detection of stroke risk factors (Papadopoulou et al., 2024; Liu et al., 2024; Huang et al., 2023). This technological synergy offers promising solutions to address the growing stroke burden through timely intervention and personalized prevention strategies.

This study aims to develop an advanced predictive model that specifically addresses the identification of individuals at elevated stroke risk, with particular emphasis on the differential impacts of various smoking behaviors. By leveraging state-of-the-art machine learning algorithms, the research seeks to enhance existing risk assessment frameworks through a more comprehensive evaluation of stroke risk determinants. The inclusion of smoking typology as a critical predictive variable represents a novel contribution to stroke prediction models, as current approaches have not sufficiently addressed the heterogeneous effects of different smoking patterns on cerebrovascular health.

The anticipated outcomes of this research are twofold: first, to provide novel insights into the relationship between smoking behaviors and stroke risk; second, to demonstrate the transformative potential of machine learning applications in clinical risk assessment. The proposed model is expected to significantly improve the accuracy of stroke risk prediction while simultaneously supporting the development of targeted prevention strategies. Ultimately, this research aims to contribute to the reduction of stroke incidence and the mitigation of its associated healthcare and socioeconomic burdens through advanced predictive analytics and personalized intervention approaches.

## 2 Materials and methods

### 2.1 Source of data

We utilized data from two major surveys: the Behavioral Risk Factor Surveillance System (BRFSS) for 2020–2021 and the National Health and Nutrition Examination Survey (NHANES).

The BRFSS, a comprehensive and nationally representative telephone survey, is jointly administered by the CDC and all U.S. states, along with participating territories. It focuses on gathering data related to behavioral risk factors.

NHANES, on the other hand, is a periodic cross-sectional survey in the US. Since the early 1960 s, the National Center for Health Statistics and the CDC have conducted it. Starting from 1999, it has been a biennial program, interviewing over 5,000 individuals per iteration. Using a complex multi - stage probability sampling method, NHANES generates nationally representative statistics for the civilian (non-institutionalized) household population. It collects a wide range of health and nutrition data, covering demographics, diet, examinations, laboratory results, and questionnaire responses. The data collection for NHANES was approved by the National Center for Health Statistics Research Ethics Review Board, with all participants’ parents or guardians providing written informed consent. Our report adheres to the Strengthening the Reporting of Observational Research in Epidemiology guidelines for presenting cross-sectional studies.

### 2.2 Study population

**FIGURE 1:**
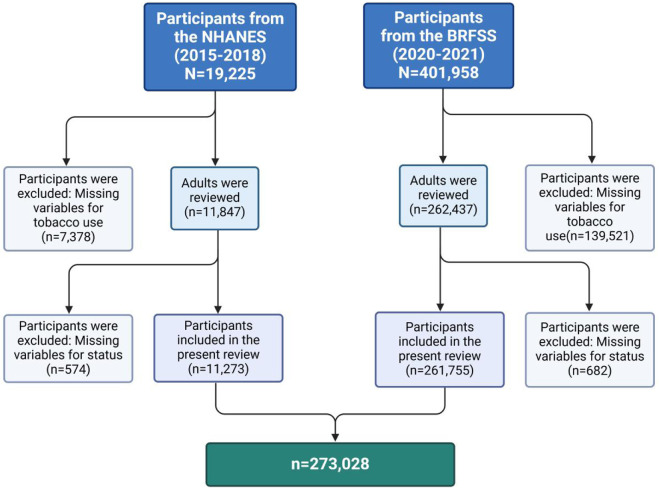
Flowchart for inclusion of study populations according to the purpose of the study.

NHANES is a cross-sectional study designed to collect data on the health and nutritional status of the U.S. population. Data is obtained through structured home interviews, physical assessments at mobile screening sites, and laboratory analyses utilizing a multistage probability sampling technique. Initially, 19,225 individuals were identified from the NHANES 2015–2018 dataset. Participants without data on tobacco use (n = 7,378) were eliminated from the study. Additionally, individuals lacking stroke status information (n = 574) were eliminated. A total of 11,273 participants were included in the final analysis. All data employed in this study are publicly available (https://www.cdc.gov/nchs/nhanes/) and have been adjusted for demographic factors for additional analysis. Furthermore, we included 401,958 participants from the 2020–2021 BRFSS. After excluding absent smoking-related data (n = 139,521) and stroke-related data (n = 682), a total of 261,755 participants were finally included (Figure 1).

### 2.3 Tobacco use assessment

The NHANES database, specifically the Smoking Cigarette Use dataset (SMQ), gathered data on cigarette intake, current use, 30-day smoking prevalence, quantity, and other smoking-related details. Participants were asked via SMQ020 if they had smoked at least 100 cigarettes in their lives and about current habits (SMQ040), and via SMQ900 if they had ever used an e-cigarette. Smoking combustible cigarettes was defined as having smoked at least 100 cigarettes in a lifetime or currently smoking daily/occasionally, while e-cigarette use was defined as any single-time use (Okafor et al., 2022).

In the 2020 BRFSS, smoking inquiries paralleled NHANES, collecting data on cigarette and e-cigarette use. BRFSS used SAS variables SMOKE100 (Column 202) to ask about lifetime smoking of at least 100 cigarettes, SMOKDAY2 (Column 203) for current smoking behavior, and ECIGARET (Column 310) for e-cigarette use. Based on these, participants were divided into four subgroups: non-smokers, exclusive combustible cigarette smokers, exclusive e-cigarette users, and dual users.

### 2.4 Stroke diagnosis

In NHANES, stroke diagnosis relied on a self-reported questionnaire (MCQ160f). Participants were asked “Has a physician or other healthcare provider ever informed you about a stroke?” with “yes” or “no” responses. A “yes” answer indicated a confirmed stroke diagnosis.

In BRFSS, stroke was diagnosed through a self-reported questionnaire (SAS variable: CVDSTRK3, Column 117). The question “Have you ever been informed that you had a stroke?” was part of the Chronic Health Conditions segment. A positive response meant the participant had experienced a stroke.

### 2.5 Covariates

To ensure consistency between NHANES and BRFSS, several covariates were considered. Age was grouped as 18–65 and 65+; sex as female or male; race as Hispanic, non-Hispanic white, non-Hispanic black, or other; education as high-school graduated or not; marital status in multiple categories; income in three brackets; exercise as yes or no. Additionally, BMI, weight, and health conditions like diabetes and total heart disease were included.

Exercise data came from different questions in BRFSS (EXERANY2: “In the last 30 days, have you engaged in”) and NHANES (PAQ746: “How frequently do you attend.”). A positive response for exercise was marked as “YES”, negative as “NO”. Diabetes was determined by the self-reported DIABETE4 question in both surveys, and total heart disease was based on self-reported SP data (CVDINFR4, CVDCRHD4) asking about heart attack, angina, or coronary artery disease.

### 2.6 Statistical analysis

Statistical analyses were conducted using R software (version 4.1.6). Owing to the complex sampling designs utilized by the NHANES and BRFSS surveys, we integrated sample weights from many study eras in our analytical approaches to accurately estimate health-related data. Through multivariate logistic regression analysis, we derived β values and 95% confidence intervals for the association between types of tobacco use and stroke incidence. The key reason for choosing multivariate logistic regression is its interpretability. Logistic regression produces clear and interpretable coefficients that represent the chances ratios associated with each predictor variable. This transparency is particularly vital in clinical and epidemiological studies, where understanding the relationship between variables and outcomes is imperative. Model 2 included adjustments for gender, age, and racial demographics, whereas Model 1 was uncorrected. A third model (Model 3) was constructed with extensive covariate adjustment for the input variables of income, marital status, education, exercise status, weight, body mass index, diabetes, and heart disease.

### 2.7 Predictive modeling and assessment

#### 2.7.1 Deterministic feature selection

Employing the Least Absolute Shrinkage and Selection Operator (LASSO) regression model, we discerned the primary predictors of stroke while accounting for the covariation among the covariates. LASSO enhances the predictive accuracy and interpretability of statistical models by integrating a penalty into the regression equation, proportional to the sum of the absolute values of the coefficients. This software effectively eliminates variables with zero coefficients using the five-fold cross-validation method with LassoCV (Python version: sklearn 0.22.1).

#### 2.7.2 ML modeling and development

We evaluated ten widely-used machine learning methods to construct and compare predictive models: XGBoost, Logistic Regression, Random Forest (RF), Gaussian Naive Bayes, LightGBM, Decision Tree (DT), k-Nearest Neighbors (KNN), CatBoost, Support Vector Machine (SVM), and Multilayer Perceptron (MLP). All models were executed using Python 3.7 and R 4.4.2. XGBoost, LightGBM, and CatBoost utilized the ‘xgboost 1.2.1′, ‘lightgbm 3.3.2′, and ‘catboost 1.0.6′ packages, respectively. Other models, including Logistic Regression, RF, Gaussian Naive Bayes, DT, KNN, SVM, and MLP, were implemented through the ‘scikit-learn 0.22.1′ package. Patients were randomly assigned to training and testing groups in an 80:20 ratio to ensure comprehensive model evaluation. For all models, hyperparameter optimization was performed via grid search combined with five-fold cross-validation to ensure fair comparison and mitigate overfitting.

#### 2.7.3 Model optimization and evaluation

Five-fold cross-validation was utilized to evaluate the model’s predictive performance and confirm its stability. To account for potential class imbalance in stroke prediction, stratified cross-validation was applied during both hyperparameter tuning and evaluation phases. The training dataset was randomly divided into five groups. During each iteration of the five-fold cross-validation, four subsets were randomly assigned as the training set, while the remaining subset functioned as the validation set. In each training phase of the model, 20% of the dataset was randomly selected from the training set to assess the model’s performance. The importance of features was assessed using the Shapley additive explanation (Shap). Features with greater absolute Shap values substantially impacted the model prediction scores. Additionally, the distribution of feature values and their relationship with model predictions were evaluated. Model performance was evaluated using seven standard metrics: Area Under the ROC Curve (AUC), Accuracy, Sensitivity (Recall), Specificity, Positive Predictive Value (PPV), Negative Predictive Value (NPV), and F1-score. The validation dataset was utilized to evaluate and compare the effectiveness of each model. The models’ ability to predict stroke was assessed using the area under the receiver operating characteristic curve for the individuals.

#### 2.7.4 Web deployment tool based on the streamlit framework

The final prediction model is included into a web application using the Streamlit Python framework to facilitate its use in a clinical setting. Upon obtaining the values of the pertinent attributes in the final model, the program can deliver the likelihood of stroke together with a graph illustrating the variations of the particular sub-items.

## 3 Results

### 3.1 Participants’ characteristics

Our combined analysis integrated data from NHANES and BRFSS, with 11,273 and 261,755 participants respectively. Key demographic and health-related differences between non-stroke and stroke groups are presented in Table 1, 2.

**TABLE 1:** Attributes of the NHANES research cohort.

Characteristic	Non-stroker (n = 10,791)	stroker (n = 482)	P-value
Characteristic			
Age (%)			<0.0001
18 ≤ age < 65	81.54	45.77	
≥65	18.46	54.23	
Sex (%)			0.4109
Male	48.16	45.89	
Female	51.84	54.11	
Income (%)			<0.0001
less than $25,000	15.25	34.41	
$25,000 to less than $50,000	25.23	28.68	
$50,000 or more	59.52	36.84	
Marital Status (%)			<0.0001
Married	53.85	49.97	
Widowed	5.47	19.04	
Divorced	9.76	14.56	
Separated	2.61	2.16	
Never married	18.73	8.40	
living with partner	9.58	5.87	
Race (%)			<0.0001
Hispanic	15.73	8.64	
Non-Hispanic White	63.01	63.83	
Non-Hispanic Black	11.24	16.92	
Other Races	10.03	10.61	
Education level (%)			<0.0001
<High school	8.53%	12.89%	
≥High school	91.47%	87.11%	
Alcohol use (%)			0.0518
Yes	63.97	56.95	
No	36.03	43.05	
BMI	29.57 ± 7.13	30.96 ± 6.83	<0.0001
Weight	83.80 ± 22.38	84.45 ± 22.14	0.6125
Diabetes (%)			<0.0001
Yes	10.58	33.71	
No	89.42	66.29	
Total heart disease (%)			<0.001
Yes	5.86	35.58	
No	94.14	64.42	
Exercise (%)			<0.001
Yes	75.96%	58.86%	
No	24.04%	41.14%	
Type of smoking			<0.0001
Non-smokers	52.92	41.06	
Only combustible cigarettes	25.25	42.14	
Only e-cigarettes	4.67	1.79	
Both combustible cigarettes and e-cigarettes	17.16	15.01	

Mean + SD, for continuous variables: the P value was calculated by the weighted linear regression model; (%) for categorical variables: the P value was calculated by the weighted chi-square test.

Abbreviations: BMI, body mass index.

**TABLE 2:** Attributes of the BRFSS study cohort.

Characteristic	Non-stroker (n = 250,890)	stroker (n = 10,865)	P-value
Characteristic			
Age (%)			<0.0001
18 ≤ age < 65	64.64%	36.44%	
≥65	35.36%	63.56%	
Sex (%)			0.4109
Male	45.14%	45.20%	
Female	54.86%	54.80%	
Income (%)			<0.0001
less than $25,000	22.41%	43.96%	
$25,000 to less than $50,000	23.77%	27.04%	
$50,000 or more	53.82%	29.00%	
Marital Status (%)			<0.0001
Married	53.13%	41.55%	
Widowed	11.09%	24.22%	
Divorced	12.94%	20.08%	
Separated	1.88%	2.85%	
Never married	17.37%	9.73%	
living with partner	53.13%	41.55%	
Race (%)			<0.0001
Hispanic	7.40%	4.01%	
Non-Hispanic White	77.44%	77.68%	
Non-Hispanic Black	7.37%	10.16%	
Other Races	7.80%	8.15%	
Education level (%)			<0.0001
<High school	6.31%	12.22%	
≥High school	93.69%	87.78%	
Alcohol use (%)			<0.0001
Yes	50.64	33.07	
No	49.36	66.93	
BMI	28.40 ± 6.40	28.95 ± 6.65	<0.0001
Weight	82.56 ± 21.07	82.93 ± 21.41	0.039
Diabetes (%)			<0.0001
Yes	12.75	31.08	
No	87.35	68.92	
Total heart disease (%)			<0.001
Yes	7.83	36.45	
No	92.17	63.55	
Exercise (%)			<0.001
Yes	76.70%	59.28%	
No	23.30%	40.72%	
Type of smoking			<0.0001
Non-smokers	74.52%	73.66%	
Only combustible cigarettes	6.06%	10.32%	
Only e-cigarettes	11.56%	6.47%	
Both combustible cigarettes and e-cigarettes	7.86%	9.55%	

Mean +SD, for continuous variables: the P value was calculated by the weighted linear regression model; (%) for categorical variables: the P value was calculated by the weighted chi-square test.

Abbreviations: BMI, body mass index.

Stroke patients were significantly older in both datasets. In NHANES, 54.23% of stroke participants were 65 or older, and in BRFSS, this percentage was 63.56% (*P* < 0.0001). Although females were more common in both stroke groups (54.11% in NHANES and 54.80% in BRFSS), the difference was not statistically significant. Income disparities were evident, with a higher proportion of lower-income stroke patients in BRFSS (43.96% vs. 34.41% in NHANES, *P* < 0.0001). Widowed individuals were more prevalent among stroke patients (19.04% in NHANES, 24.22% in BRFSS, *P* < 0.0001). Non-Hispanic Black participants were more common in stroke groups compared to non - stroke groups (16.92% in NHANES and 10.16% in BRFSS, *P* < 0.0001). Non-stroke groups had higher educational attainment, with over 85% of stroke patients having at least a high-school education in both datasets (*P* < 0.0001).

Regarding health and lifestyle, stroke patients had a higher BMI (30.96 ± 6.83 in NHANES and 28.95 ± 6.65 in BRFSS, *P* < 0.0001). Diabetes and heart disease were more common in stroke patients (33.71% and 35.58% in NHANES, 31.08% and 36.45% in BRFSS, *P* < 0.001). Stroke patients exercised less (58.86% in NHANES, 59.28% in BRFSS, *P* < 0.001) and were more likely to be current combustible cigarette smokers (42.14% in NHANES, 10.32% in BRFSS, *P* < 0.0001).

### 3.2 The association between cigarette use and stroke

**FIGURE 2:**
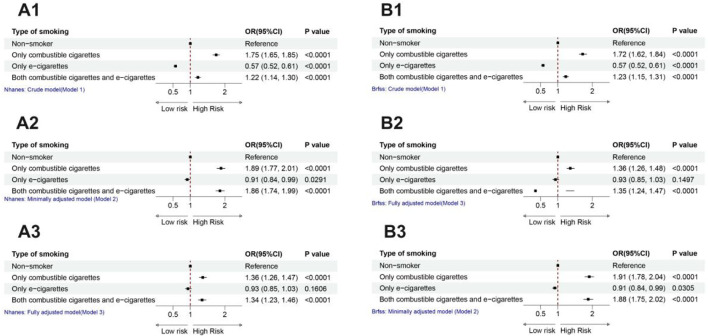
Forest plot of the relationship between cigarette use and stroke.

Figure 2 demonstrates the association between tobacco use patterns and stroke risk in individuals aged 20 and older, analyzed using three statistical models. Model 1 is unadjusted, Model 2 adjusts for demographic factors, and Model 3 fully adjusts for confounders such as age, sex, race, and comorbidities.

In Model 3, exclusive use of combustible cigarettes (β: 1.34–1.36, 95% CI: 1.26–1.47, *P* < 0.0001) and dual use of combustible cigarettes with e-cigarettes (β: 1.34, 95% CI: 1.23–1.46, *P* < 0.0001) were strongly associated with increased stroke risk. These results indicate that combustible cigarette use, whether alone or combined with e-cigarettes, significantly elevates stroke risk after adjusting for confounders.

In contrast, exclusive e-cigarette use showed no significant association with stroke risk in Model 3 (*P* > 0.05), suggesting a distinct risk profile compared to combustible cigarettes. However, in Model 2, all smoking modalities, including e-cigarette use, were significantly associated with stroke risk, highlighting the influence of demographic factors on these associations.

### 3.3 A prediction model to evaluate the stroke risk

The selection of variables included in the LASSO regression model was based on a combination of clinical relevance, prior literature, and statistical considerations. Specifically, we conducted an extensive literature review on known risk factors for stroke and included variables that have been widely recognized as relevant predictors (e.g., age, BMI, smoking status, hypertension, diabetes, and other cardiovascular risk factors) (Fu et al., 2024; Dutta et al., 2024; Notley et al., 2025; Hjort et al., 2024; Jumbe et al., 2025). Additionally, we ensured that all selected variables were available in both datasets (NHANES and BRFSS) to maintain consistency across analyses. LASSO regression was employed due to its ability to perform automatic variable selection by shrinking less important coefficients to zero, thereby reducing model complexity and improving interpretability. By applying 5-fold cross-validation, we optimized the regularization parameter (λ) to achieve a balance between model performance and feature sparsity.

**FIGURE 3:**
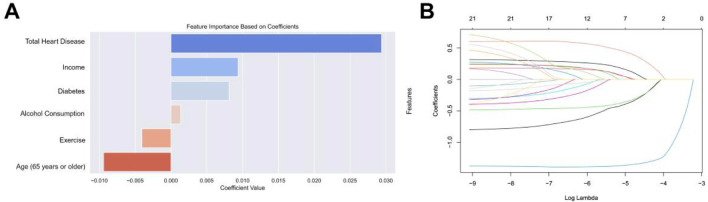
**(A)** Selection of features with non-zero coefficients and their coefficients using the LASSO regression method. **(B)** The impact of the penalty coefficient λ on the weight coefficients of each independent variable is represented on the horizontal axis as λ and on the vertical axis as the weight coefficients, with distinct colors indicating the weight coefficients of individual independent variables.

We used baseline characteristics for stroke risk prediction. Through the LASSO method for feature selection, six key predictors were identified: ‘age’, ‘income’, ‘exercise’, ‘alcohol consumption’, ‘diabetes’, and ‘total heart disease’. These were determined to be the most valuable for building the predictive model using LASSO regularization and five-fold cross-validation. Figure 3 shows the coefficients for each variable in the LASSO model.

### 3.4 Model explanation

**FIGURE 4:**
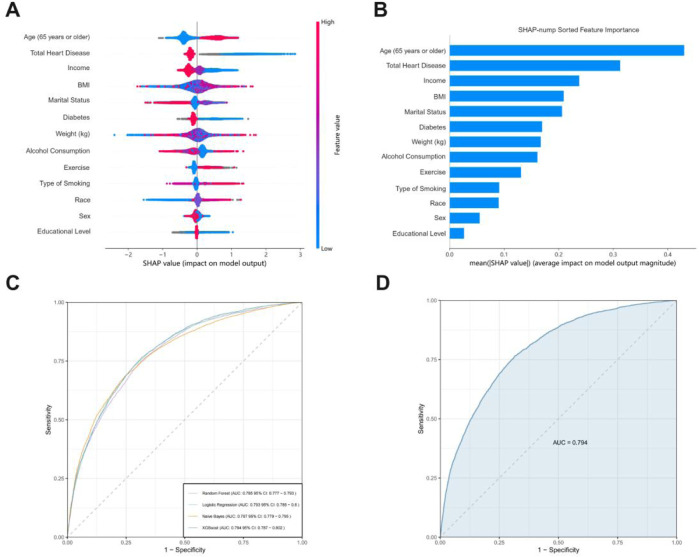
**(A)** The horizontal coordinates indicate the magnitude of the SHAP value, with positive values representing the positive contribution of the variable to a positive stroke outcome and negative values the opposite; the color ranges from blue to red to characterize the low to high values of the variable in order. **(B)** SHAP evaluations of the XGBoost algorithm for forecasting adverse outcomes in stroke patients. **(C)** The mean AUC performance of four machine learning models evaluated using five-fold external cross-validation. **(D)** ROC curve examination of the XGBoost algorithms for predicting stroke risk in the external test set.

We compared XGBoost, logistic regression, RF, and Gaussian Naive Bayes (Figure 4). While the initial visualization focuses on four representative models (linear vs nonlinear, parametric vs non-parametric), Supplementary Figure S1 in the Supplement provides complete ROC comparisons across all ten evaluated models. The results show that XGBoost has the best predictive ability (AUC = 0.794) among the primary candidates, though LightGBM achieved comparable performance (Validation AUC = 0.793) as detailed in Supplementary Table S1. Notably, simpler models like Logistic Regression (AUC = 0.785) demonstrated adequate discrimination, whereas KNN showed significant overfitting with a 25.3% AUC drop from training (0.930) to validation (0.742), likely due to local noise sensitivity in high-dimensional space. Following this, we utilized XGBoost to build a clinical prediction model given its optimal balance between performance and computational efficiency.

We compared the difference in AUC using Delong’s nonparametric method with MedCalc version 19.6 (https://www.medcalc.org), progressively reducing the features of the selected ML model until a significant decrease in AUC was observed. We illustrated the predictive influence of the variables on the outcomes using SHAP plots. The impact of variables on outcomes can be visually assessed through the amplitude of SHAP values (shown by color variations) and the trend along the horizontal axis (likelihood of an adverse event). In the image on the right, older individuals (shown in red) have a higher likelihood of bad prognosis compared to younger individuals (represented in blue). Individuals with a high BMI (red) are prone to experience a poor outcome as stroke patients (right panel).

### 3.5 Convenient application for clinical utility

**FIGURE 5:**
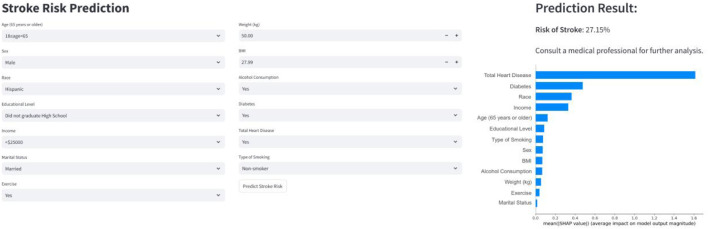
Web-based calculator predicts stroke risk.

Figure 5 illustrates the integration of the final prediction model into a web application to improve its practical utility in a clinical environment. In addition to risk prediction, SHAP summary plots were generated to illustrate the contribution of each feature to the decision-making process. Features highlighted in red indicate higher risk, while features highlighted in blue indicate lower risk. The web application can be accessed at the URL: http://10.50.1.58:8502.

## 4 Discussion

This study demonstrates a strong association between tobacco consumption and the risk of stroke. Epidemiological studies consistently demonstrate a substantial association between smoking behaviors and increased stroke incidence, with smokers experiencing a much higher risk than non-smokers (Wang X. et al., 2024; Feigin et al., 2024; Shi et al., 2023; Elser et al., 2023). Our machine learning framework extended these observations by identifying nonlinear risk thresholds. Meanwhile, this risk extends to both active smokers and those exposed to secondhand smoke (Lin et al., 2016; Lu et al., 2024), emphasizing the need for targeted public health interventions to reduce smoking rates. The intricate relationship between smoking and stroke highlights the importance of addressing tobacco use as a critical component of stroke prevention strategies.

The molecular mechanisms through which smoking contributes to stroke risk involve a variety of harmful constituents present in tobacco smoke, many of which are toxic and carcinogenic (Adler et al., 2023; Jang et al., 2024; Liang et al., 2022). Key components such as carbon monoxide and nicotine induce endothelial dysfunction and promote a pro-inflammatory state (Rezk-Hanna et al., 2019; Wang et al., 2023; Belkin et al., 2023), leading to vascular damage (Whitehead et al., 2021). Additionally, oxidative stress resulting from the inhalation of free radicals damages vascular endothelium, exacerbating atherosclerotic processes and increasing thrombogenic potential (Qin et al., 2020; Higashi, 2023). Notably, the accumulation of these detrimental substances impairs the healing of vascular injuries, further predisposing individuals to the development of cerebrovascular diseases (Siegel et al., 2022). These mechanisms align with our findings of a significantly increased stroke risk in traditional cigarette users. E-cigarettes became commercially available in the US in 2007 and have since gained widespread popularity among both adults and adolescents as they are generally considered to produce fewer toxins than traditional cigarettes (Sood et al., 2018; Goniewicz et al., 2017). However, emerging evidence suggests that they may exert cerebrovascular toxicity via nicotine-induced oxidative stress and inflammatory cytokine release (Crotty Alexander et al., 2018; Benowitz and Fraiman, 2017),and adversely affect the circulatory system through mechanisms such as increased heart rate and blood pressure, endothelial dysfunction, and accelerated platelet aggregation (Qasim et al., 2017; Goniewicz et al., 2014). Preclinical evidence shows that chronic e-cigarette exposure compromises blood-brain barrier (BBB) integrity and exacerbates ischemic injury, comparable to traditional smoking (Kaisar et al., 2017).

To enhance clinical utility, we developed a predictive model utilizing baseline characteristics as potential predictors of stroke risk. LASSO identified six linearly associated predictors, whereas SHAP revealed BMI as a critical nonlinear factor indicating that obesity elevated stroke risk through different mechanisms. While age consistently emerged as the strongest predictor reflecting cumulative vascular damage, BMI’s absence in LASSO contrasted with its SHAP prominence, highlighting threshold effects captured by XGBoost. This divergence underscores the complementary strengths of LASSO and SHAP, advocating for their joint use in heterogeneous risk profiling.

The strengths of our research are rooted in its comprehensive approach, utilizing multiple machine learning algorithms to determine the most effective model for stroke prediction. Notably, the severe class imbalance (4.16% stroke cases) was addressed through XGBoost’s scale_pos_weight parameter and weighted logistic regression, which improved model sensitivity while maintaining specificity. Additionally, we developed an online predictive platform, enhancing accessibility for clinicians. However, conventional accuracy metrics may overestimate clinical utility given this imbalance, as evidenced by the discrepancy between training set performance (XGBoost F1 = 0.808) and validation metrics (F1 = 0.837). Subsequent research should pursue multi-center validations incorporating advanced sampling techniques and composite metrics like AUC-PR to better handle skewed distributions.

However, we are also acutely aware of several limitations in our study. First, the extreme class imbalance (95.84% non-stroke cases) and potential biases from self-reported data may affect model accuracy and generalizability, particularly for low-prevalence predictors like e-cigarette use. Second, the cross-sectional design precludes causal inference and cannot account for reverse causality. Third, our analysis revealed no significant association between exclusive e-cigarette use and stroke risk in fully adjusted models, which may reflect methodological limitations such as insufficient capture of long-term cumulative exposure, underreporting of dual use with combustible products, or lack of granularity on e-cigarette consumption patterns. Additionally, the biological latency of cerebrovascular damage from e-cigarettes may exceed our observational timeframe, suggesting potential long-term harm that warrants further investigation using available long-term clinical and animal data. These findings underscore the need for comprehensive tobacco cessation strategies prioritizing complete nicotine abstinence over e-cigarette use alone.

In terms of future research directions, multi-center validations across diverse countries and ethnic groups are essential to confirm the generalizability of our model and findings. Large-scale prospective studies incorporating repeated exposure measurements are necessary to establish temporal precedence between e-cigarette use and stroke outcomes, thereby clarifying causal relationships. Long-term follow-up of participants will provide more precise insights into the temporal progression of stroke development.

In summary, our predictive model represents a significant advancement in stroke risk assessment, providing healthcare professionals with a robust tool for informed decision-making. The potential for widespread application in clinical settings holds promise for improving stroke prevention strategies, ultimately leading to better health outcomes for individuals at risk. We look forward to the continued exploration of this model’s utility across diverse clinical scenarios and its role in enhancing patient care.

## 5 Conclusion

This study establishes that distinct tobacco consumption patterns differentially elevate stroke risk, with combustible tobacco products demonstrating the strongest association. Through machine learning-driven feature selection, we developed and validated a clinical prediction tool achieving in stroke risk stratification. The integration of this model into an open-access web platform enables real-time, individualized stroke risk assessment, offering clinicians a practical tool for targeted intervention strategies. These findings underscore the importance of smoking cessation in stroke prevention and provide a scalable solution for risk stratification in high-risk populations. Future studies should focus on multi-center validation and further exploration of dose-dependent effects of tobacco use to enhance the generalizability and precision of the model.

## Data Availability

The raw data supporting the conclusions of this article will be made available by the authors, without undue reservation.

## Abbreviations

ML, machine learning; NHANES, National Health and Nutrition Examination Survey; BRFSS, Behavioral Risk Factor Surveillance System; ORs, electronic cigarettes, e-cigarettes; odds ratios; CIs, confidence intervals; RF, random forest; AUC, area under the subject-operating characteristic curve; AI, artificial intelligence; DL, deep learning; LASSO, Least Absolute Shrinkage and Selection Operator; BMI, body mass index; LightGBM, Light Gradient Boosting Machine; DT, Decision Tree; KNN, K-Nearest Neighbors; CatBoost, Categorical Boosting; SVM, Support Vector Machine; MLP, Multi-Layer Perceptron.
