# Supplementary material for: Cross-sectional study on smoking types and stroke risk: development of a predictive model for identifying stroke risk
Source: Front Physiol. 2025 Mar 24;16:1528910. doi: 10.3389/fphys.2025.1528910 (PMC11973365; doi:10.3389/fphys.2025.1528910)
Supplement: Supplementary file 1 [file Supplementaryfile1.docx]

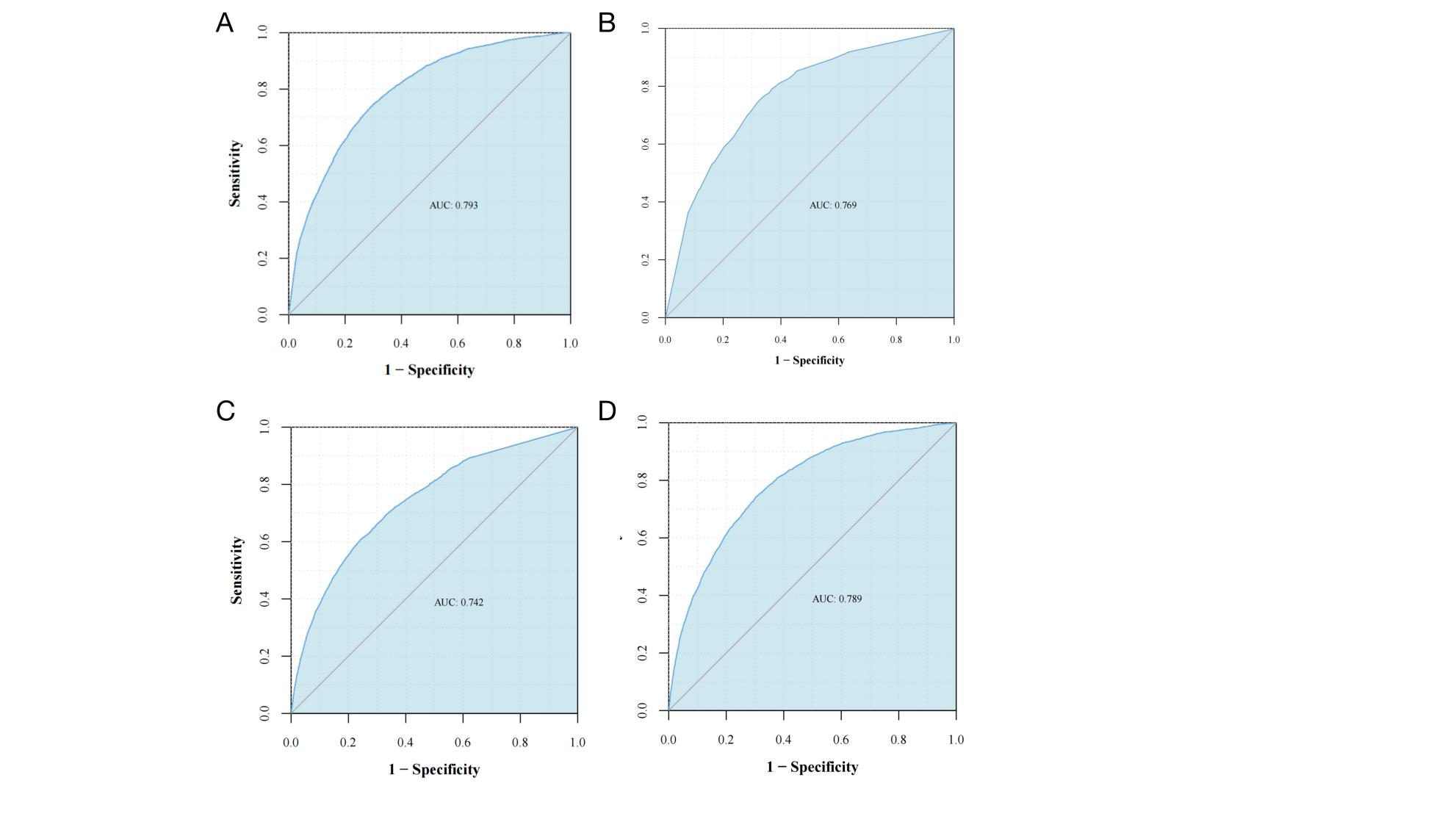


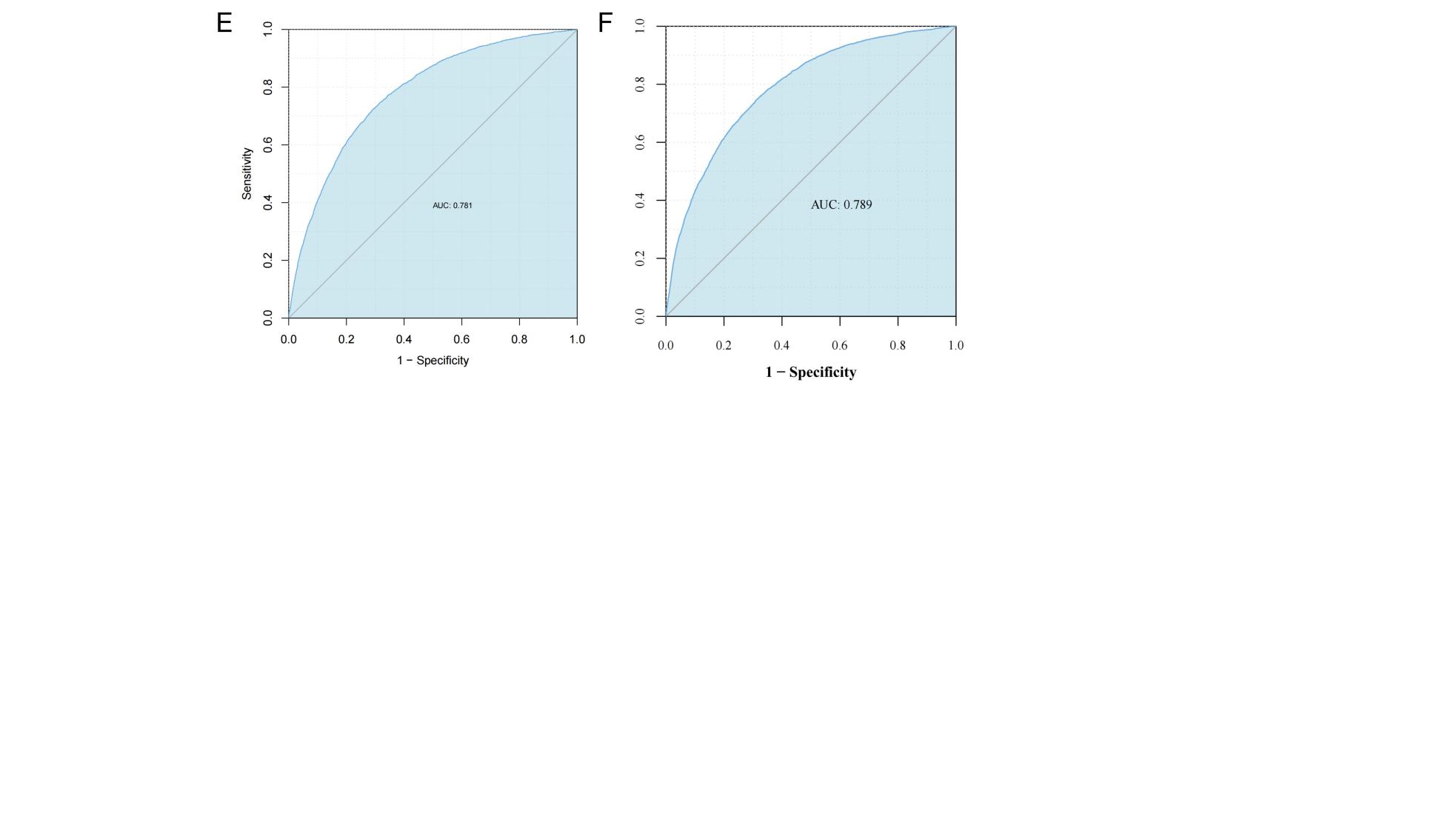


****Figure S1**.** ROC curve analysis and AUC performance of six machine learning models for stroke risk prediction in the external test set. (A) LightGBM, (B) DT, (C) KNN, (D) CatBoost, (E) SVM, (F) MLP.

| **Table S1** | **Performance comparison of machine learning models on stroke risk prediction** |  |  |  |  |  |  |
| --- | --- | --- | --- | --- | --- | --- | --- |
| Training Set |  |  |  |  |  |  |  |
| Models | AUC | Accuracy | Sensitivity | Specificity | PPV | NPV | F1 score |
| KNN | 0.930 | 0.782 | 0.938 | 0.775 | 0.155 | 0.997 | 0.266 |
| CatBoost | 0.841 | 0.760 | 0.780 | 0.740 | 0.749 | 0.771 | 0.764 |
| LightGBM | 0.818 | 0.740 | 0.725 | 0.756 | 0.749 | 0.732 | 0.737 |
| SVM | 0.809 | 0.786 | 0.791 | 0.672 | 0.707 | 0.763 | 0.198 |
| MLP | 0.801 | 0.726 | 0.739 | 0.714 | 0.720 | 0.733 | 0.170 |
| XGBoost | 0.798 | 0.726 | 0.729 | 0.713 | 0.906 | 0.409 | 0.808 |
| RF | 0.779 | 0.712 | 0.744 | 0.679 | 0.699 | 0.726 | 0.721 |
| GNB | 0.776 | 0.707 | 0.687 | 0.726 | 0.714 | 0.700 | 0.700 |
| LG | 0.785 | 0.712 | 0.674 | 0.749 | 0.728 | 0.698 | 0.700 |
| DT | 0.792 | 0.712 | 0.772 | 0.709 | 0.105 | 0.986 | 0.184 |
|  |  |  |  |  |  |  |  |
| Validation Set |  |  |  |  |  |  |  |
| Models | AUC | Accuracy | Sensitivity | Specificity | PPV | NPV | F1 score |
| KNN | 0.742 | 0.751 | 0.609 | 0.756 | 0.095 | 0.979 | 0.165 |
| CatBoost | 0.789 | 0.732 | 0.697 | 0.733 | 0.099 | 0.983 | 0.173 |
| LightGBM | 0.793 | 0.718 | 0.717 | 0.728 | 0.984 | 0.098 | 0.830 |
| SVM | 0.781 | 0.792 | 0.607 | 0.800 | 0.113 | 0.980 | 0.191 |
| MLP | 0.789 | 0.720 | 0.710 | 0.720 | 0.096 | 0.983 | 0.170 |
| XGBoost | 0.794 | 0.727 | 0.728 | 0.720 | 0.984 | 0.100 | 0.837 |
| RF | 0.785 | 0.684 | 0.758 | 0.681 | 0.091 | 0.985 | 0.162 |
| GNB | 0.787 | 0.725 | 0.704 | 0.726 | 0.098 | 0.983 | 0.171 |
| LG | 0.793 | 0.746 | 0.693 | 0.749 | 0.104 | 0.983 | 0.181 |
| DT | 0.769 | 0.703 | 0.713 | 0.703 | 0.092 | 0.983 | 0.162 |
|  |  |  |  |  |  |  |  |
| Notes: AUC = Area Under the ROC Curve; PPV = Positive Predictive Value; NPV = Negative Predictive Value; TP = True Positive; TN = True Negative; FP = False Positive; FN = False Negative; KNN = k-Nearest Neighbors; CatBoost = Categorical Boosting; LightGBM = Light Gradient Boosting Machine; SVM = Support Vector Machine; MLP = Multilayer Perceptron; XGBoost = eXtreme Gradient Boosting; RF = Random Forest; GNB = Gaussian Naive Bayes; LG = Logistic Regression; DT = Decision Tree. | | | | | | | |
|  |  |  |  |  |  |  |  |
|  |  |  |  |  |  |  |  |
|  |  |  |  |  |  |  |  |
|  |  |  |  |  |  |  |  |
|  |  |  |  |  |  |  |  |

**Table S1.** Performance comparison of machine learning models on stroke risk prediction.
